# The importance of considering community-level effects when selecting insecticidal malaria vector products

**DOI:** 10.1186/1756-3305-4-160

**Published:** 2011-08-12

**Authors:** Gerry F Killeen, Fredros O Okumu, Raphael N'Guessan, Marc Coosemans, Adedapo Adeogun, Sam Awolola, Josiane Etang, Roch K Dabiré, Vincent Corbel

**Affiliations:** 1 Ifakara Health Institute, Biomedical & Environmental Thematic Group, PO Box 53, Ifakara, Morogoro, United Republic of Tanzania; 2 Liverpool School of Tropical Medicine, Vector Group, Pembroke Place, Liverpool L3 5QA, UK; 3 London School of Hygiene and Tropical Medicine, Disease Control and Vector Biology Unit, Keppel Street, WCIE 7HT, London UK; 4 Centre de Recherche Entomologique de Cotonou (CREC), Laboratoire Nationale, Ministère de la Santé, Cotonou 06 BP 2604, Benin; 5 Institute of Tropical Medicine Antwerp, Department of Parasitology, Nationalestraat 155, B-2000 Antwerpen, Belgium; 6 Department of Biomedical Sciences, Faculty of Pharmaceutical, Veterinary and Biomedical Sciences, University of Antwerp, Universiteitsplein 1, B-2610 Antwerpen, Belgium; 7 Public Health Division, Nigerian Institute of Medical Research, Yaba, Lagos; 8 Organisation de Coordination pour la lutte contre les Endémies en Afrique Centrale (OCEAC), BP. 288, Yaoundé, Cameroun; 9 Faculty of Medicine and Pharmaceutical Sciences, University of Douala, P.O.Box 2701 Douala, Cameroon; 10 Institut de Recherche en Sciences de la Santé (IRSS)/Centre Muraz (CM), 01 BP 390, Bobo-Dioulasso, Burkina Faso; 11 Institut de Recherche pour le Développement (IRD), Maladies Infectieuses et Vecteurs, Ecologie, Génétique, Evolution et Contrôle (MIVEGEC), UM1-CNRS 5290-IRD 224, Centre de Recherche Entomologique de Cotonou (CREC), Cotonou, Benin

## Abstract

**Background:**

Insecticide treatment of nets, curtains or walls and ceilings of houses represent the primary means for malaria prevention worldwide. Direct personal protection of individuals and households arises from deterrent and insecticidal activities which divert or kill mosquitoes before they can feed. However, at high coverage, community-level reductions of mosquito density and survival prevent more transmission exposure than the personal protection acquired by using a net or living in a sprayed house.

**Methods:**

A process-explicit simulation of malaria transmission was applied to results of 4 recent Phase II experimental hut trials comparing a new mosaic long-lasting insecticidal net (LLIN) which combines deltamethrin and piperonyl butoxide with another LLIN product by the same manufacturer relying on deltamethrin alone.

**Results:**

Direct estimates of mean personal protection against insecticide-resistant vectors in Vietnam, Cameroon, Burkina Faso and Benin revealed no clear advantage for combination LLINs over deltamethrin-only LLINs (P = 0.973) unless both types of nets were extensively washed (Relative mean entomologic inoculation rate (EIR) ± standard error of the mean (SEM) for users of combination nets compared to users of deltamethrin only nets = 0.853 ± 0.056, P = 0.008). However, simulations of impact at high coverage (80% use) predicted consistently better impact for the combination net across all four sites (Relative mean EIR ± SEM in communities with combination nets, compared with those using deltamethrin only nets = 0.613 ± 0.076, P < 0.001), regardless of whether the nets were washed or not (P = 0.467). Nevertheless, the degree of advantage obtained with the combination varied substantially between sites and their associated resistant vector populations.

**Conclusion:**

Process-explicit simulations of community-level protection, parameterized using locally-relevant experimental hut studies, should be explicitly considered when choosing vector control products for large-scale epidemiological trials or public health programme procurement, particularly as growing insecticide resistance necessitates the use of multiple active ingredients.

## Background

Insecticide treatment of nets, curtains or walls and ceilings of houses represent the primary means for malaria prevention worldwide [1,2]. Direct personal protection of individuals and households arises from deterrent and insecticidal activities which divert or kill mosquitoes before they can feed [3,4]. World Health Organization Pesticide Evaluation System (WHOPES) Phase II trials to evaluate such measures in experimental huts therefore quantify the efficacy of such measures in terms of the proportional reduction in the number of blood-fed mosquitoes caught or found dead within the huts [5]. However, at high coverage, community-level suppression of transmission is thought to be more important than personal protection because the impact of coverage among neighbours upon mosquito density and survival prevents more malaria transmission than using a net or living in a sprayed house [6]. Indeed such reasoning underlies the prioritization of universal coverage of all age groups with either long-lasting insecticidal nets (LLINs) or indoor residual spraying (IRS) as a target for malaria-endemic African countries [7,8].

WHOPES approves individual products based on assessments of safety, efficacy and operational acceptability, as well as developing specifications for quality control and international trade [5]. However, choosing between individual products, or combinations thereof, requires consideration of a variety of other important factors such as cost, durability, potential to mitigate the emergence of insecticide resistance and expected impact upon disease transmission. While current guidelines correspondingly emphasize the need to conduct large-scale field trials of promising products at high coverage rates, no explicit recommendation has been made about how to evaluate and compare alternative insecticidal products in terms of their overall potential to control malaria. Standardized experimental hut methodologies [5,9] do quantify personal protection in terms of proportional reduction of blood feeding, and the insecticidal impact that predominantly underlies communal protection [10] in terms of the proportion of mosquitoes killed, but no specific guidelines exist for relating these important properties to expected overall impact.

We therefore suggest that explicitly simulating expected community-level impacts of each of a range of products, using data derived from comparative experimental hut trials, may be a useful intermediate planning step between assessing personal protective efficacy and proceeding to large scale trials, or even directly to procurement for public health programmes. Here we use a process-explicit simulation of malaria transmission, which distinguishes the impact of deterrent, as well as fast and slow-acting insecticidal activities [10], to re-examine the outcomes of recently published Phase II trials comparing a new LLIN [11] product which supplements deltamethrin with piperonyl butoxide (PermaNet^® ^3.0) with a similar conventional product by the same manufacturer (Vestergaard Frandsen SA, Switzerland), but relying on deltamethrin alone (PermaNet^® ^2.0) [12-14].

## Results

### Deltamethrin plus piperonyl butoxide combination LLIN versus deltamethrin-only LLIN

**Figure 1:**
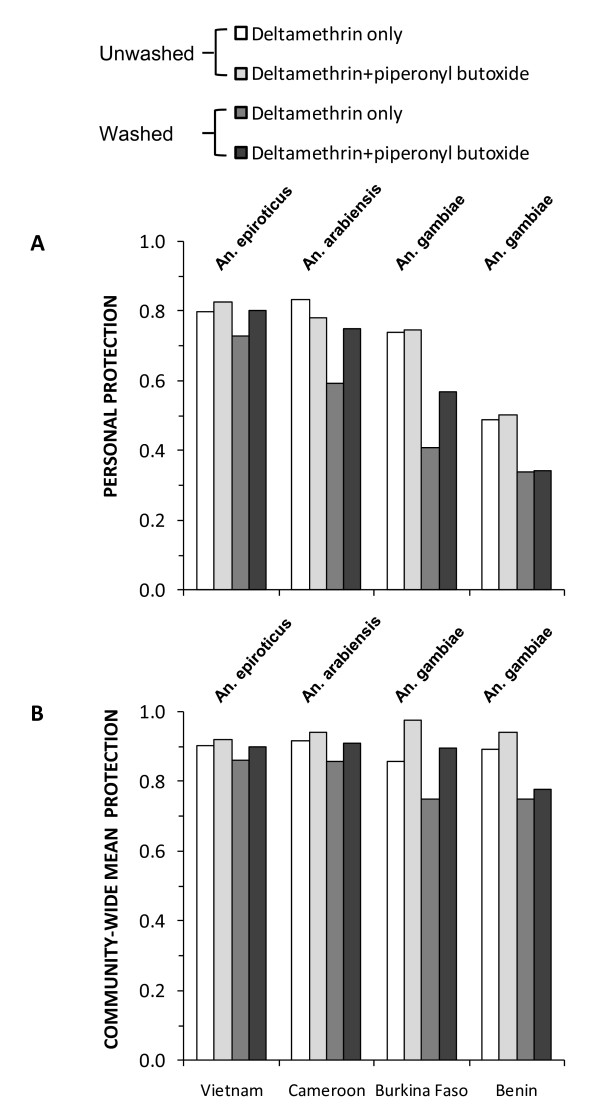
**Directly estimated direct personal protection (A) and predicted mean overall protection assuming 80% use (B) against malaria transmission exposure for fresh and washed specimens of deltamethrin plus piperonyl butoxide combination LLINs and for deltamethrin-only LLINs, based on experimental hut field trials with insecticide-resistant vector populations from Vietnam **[32], **Benin **[14], **Cameroon and Burkina Faso **[13].

**Figure 2:**
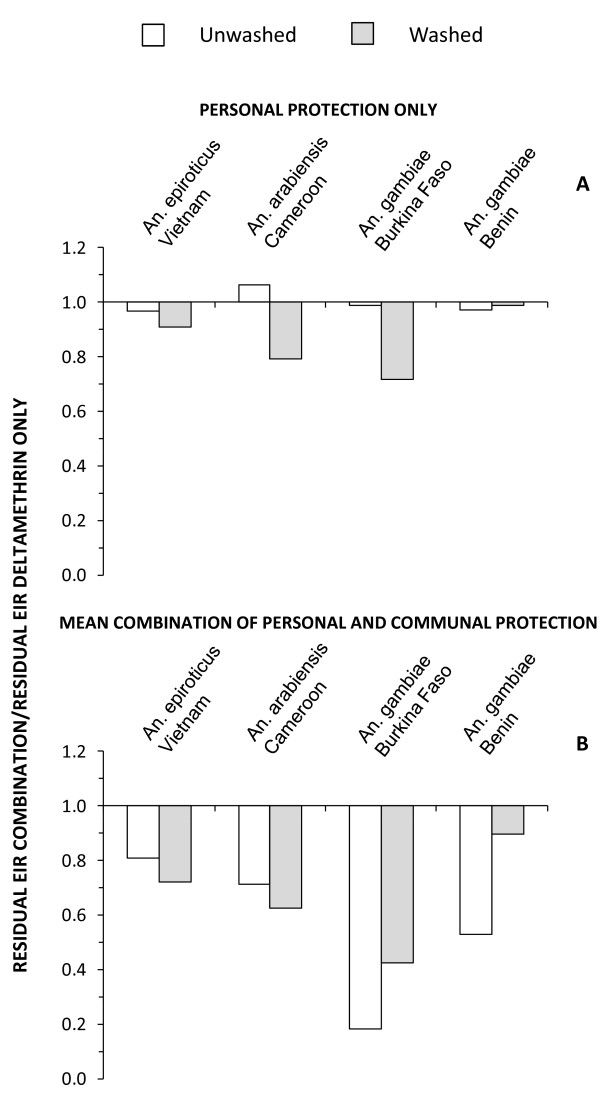
**Relative residual entomological inoculation rate for (A) individual users at negligible net coverage and (B) the average community member at high coverage (80% use) for deltamethrin plus piperonyl butoxide combination LLINs compared to those with deltamethrin-only LLINs, based on experimental hut field trials with insecticide-resistant vector populations from Vietnam **[32], **Benin **[14], **Cameroon and Burkina Faso **[13].

Review of direct field estimates of personal protection through experimental hut trials (Figure 1A) revealed no clear advantage of the new combination LLIN, relative to the deltamethrin-only product, unless both had been washed extensively. This trend is more clearly illustrated in Figure 2A which presents the EIR experienced by a combination net user relative to that experienced by a user of a deltamethrin-only net. EIR for users of combination nets was not lower than for users of deltamethrin-only nets (P = 0.973) unless they had been washed (mean relative EIR ± standard error of the mean (SEM) = 0.853 ± 0.056, P = 0.008). In all cases, washing attenuated personal protection but the direct protective efficacy of the combination product proved to be more durable (Figure 1A, 2A). Such consistently better personal protection with the combination product when washed might suggest this as the preferred LLIN. However, the case for making such a choice becomes less clear when one considers the lack of a clear advantage of the combination product when freshly distributed.

By contrast, simulations of anticipated impact at 80% use across entire communities (Figure 1B) revealed consistently equivalent-or-greater community-wide mean overall (personal plus communal) protection with the new combination product across all four sites, regardless of whether these LLINS were washed or not. Conventional plots of overall protection (Figure 1B) under-represent the importance of differences between such products, especially when considering total communal and personal protection. This is because the incremental impact acts on the remaining residual transmission once high coverage is achieved rather than that which occurred at baseline. Comparing the level of residual transmission that can be expected with the two LLIN products when used at high coverage levels reveals a consistent, sometimes sizable advantage (Figure 2B). The predicted mean EIR in communities with high coverage of combination nets, compared with those using deltamethrin only nets (Figure 2) is reduced overall (mean relative EIR ± SEM = 0.613 ± 0.076, P < 0.001), regardless of whether the nets were washed or not (P = 0.467).

The advantage of the combination product is most clear for the *An. gambiae *population in Burkina Faso with high frequencies of the *kdr *L1014F resistance allele but no known metabolic resistance, where residual transmission expected with high coverage of deltamethrin-only LLINs can be further reduced by a further 2- and 5- fold if this product is replaced with the combination product for washed and fresh nets, respectively. By contrast, it is noteworthy that the benefit of using the new combination LLIN is appreciable for unwashed nets at the Benin site, where the least personal and communal protection is obtained with its conventional predecessor, but that this advantage essentially disappears following washing. This suggests that such combination LLINs may have limited potential for tackling malaria transmission in this areas where combined *kdr *and metabolic resistance mechanisms both occur in the local *An. gambiae *population.

## Discussion

PermaNet^® ^3.0 was developed for increased efficacy against pyrethroid-resistant malaria vectors, when compared to conventional LLINs with a single pyrethroid active ingredient. In the example described in Figures 1 and 2, these simulations seem to suggest a far less ambiguous choice between two LLIN products from the same manufacturer than would be obvious from experimental hut estimates of personal protection and mosquito mortality. At high coverage, the community-level protection provided by LLINs is always far greater than direct personal protection because the former reflects the accumulated effects of repeated risk of exposure of mosquitoes to protected humans over the long lifespan they require to acquire, mature and then transmit sporogonic-stage parasites [6]. Apparently modest differences between products in personal protection can therefore result in far larger and more obvious differences in the community-level protection they can provide.

The fact that PermaNet^® ^3.0 not only contains the synergist PBO on the roof, but also higher content of the pyrethroid deltamethrin than PermaNet^® ^2.0 may well contribute to the generally higher performance of the combination nets, especially after washing. Nevertheless, even within this small set of four sites, a wide range of predicted advantages of the combination net product is apparent: while the results from Burkina Faso are extremely encouraging, those from Benin with washed nets merit equal consideration. Ultimately, this analysis merely represents an example which should not be over-interpreted in its own right. However, such analyses may stimulate more locally intensive and geographically extensive field investigations, complemented with appropriate modelling analyses, of how best to tackle insecticide resistance and malaria transmission with vector control products combining multiple active ingredients.

The simulations described in Figures 1 and 2 complement conventional analyses and strengthen our ability to interpret the data in a balanced way. While such simulations are by no means a substitute for actually measuring these crucially important community-level benefits in the field, to do so necessitates trials in which the replicated experimental units are not individual humans but rather entire communities that are large enough to minimize the blending of treatment impacts across space as a result of mosquito dispersal [15,16]. To our knowledge, no such large-scale trials that unambiguously and directly compare the epidemiological impact of alternative products or product combinations have been completed [1,2,17]. Furthermore, comparative epidemiological trials on such enormous scales necessarily cost millions of dollars, take several years to complete, and can therefore only be undertaken for a carefully selected minority of the most promising or controversial products and product combinations. In the meantime, descriptive reviews [2,17,18] remain the only empirical experimental evidence available with which to compare the large-scale effectiveness of alternative products. Faced with such a paucity of empirical evidence, the process of selecting products for procurement by public health programmes, and even for large-scale epidemiological trials, can only be enhanced by critical assessment of such simulation results.

It should be noted, however, that such simulations of efficacy have significant limitations [10] and represent just one of many criteria to consider for selecting products for further trials or even for direct public heath application. All mathematical models represent simplified conceptualizations of complex real-world processes that rely on imprecise field estimates or educated guesses to set input parameter values. Such models are therefore, by definition, inaccurate to some degree and some of the greatest mistakes in the history of malaria control policy formulation [19] have been based on upon direct interpretation of model outputs at face value [20,21] that subsequently proved somewhat unrealistic [22]. Like any other simulation modelling outputs, these predictions merely represent educated guesses, constituting evidence for plausibility but not probability for the predicted outcomes.

The most obvious limitation of this particular analysis is that it does not consider the possibility that the mortality and diversion parameters measured in experimental huts may change as coverage with a specific product rises and resistance traits against relevant active ingredients become increasingly frequent. Also, both the model and experimental hut surveys that support it, assume that resistance traits are consistently expressed as a fixed phenotype across all ages but this may not always be the case for resistant vector populations [23,24]. In this particular comparison, the predicted advantages of PermaNet^® ^3.0 cannot be unambiguously attributed to either the addition of the PBO synergist or the higher detamethrin concentration so the potential of this product to either retard or accelerate the emergence of pyrethroid resistance remains unclear. However, it is worth noting that the manufacturer of this particular product claims only that PermaNet^® ^3.0 has greater efficacy than its predecessor and these simulations do strengthen that claim.

Beyond predicted efficacy and potential to mitigate insecticide resistance, a number of equally important factors must also be considered when selecting products for malaria vector control, notably as cost, safety, acceptability, durability of efficacy in the field and local delivery system options. Simulations such as those presented here are therefore intended to complement rather than replace existing criteria for selecting insecticidal products for malaria vector control. We therefore emphasize that this analysis is not prescriptive nor are we recommending any of the particular products described here: model predictions should merely be considered as part of the evidence base to be weighed up by malaria control programmes, their funding partners, their technical advisors and the research community that implements large scale epidemiological trials.

## Conclusion

Simulations from models such as this one [10], and also a number of recent alternative formulations [4,25,26], may well be useful for anticipating the likely outcome of large-scale trials and public health programmes. Indeed such models may be particularly useful for examining the potential of products with non-deterrent, slow-acting, contact insecticides which may provide little personal protection but excellent community-level protection [4,10,26]. Examples of such products include entomopathogenic fungi [27], chlorfenapyr [28], bendiocarb [29], chlorpyrifos methyl [30] and even pyrethroid-based LLINs that have been depleted of insecticide after several years of use [31]. We therefore conclude that model predictions for community-level protection should be explicitly considered as part of the evidence base when choosing from a range of potential products for procurement by malaria control programmes, or even further large-scale trials, particularly as growing insecticide resistance necessitates the use of multiple active ingredients.

## Methods

### Phase II experimental hut evaluations

All four trials referred to here were conducted according to World Health Organization guidelines [5] using standard experimental hut designs [9] in areas where transmission is mediated by vector populations with documented high frequencies of resistance to pyrethroids. *Anopheles epiroticus *in Van Duc A village, Bac Lieu province in the Mekong Delta of southern Vietnam are known to be resistant to deltamethrin, alpha-cypermethrin, etofenprox and cyfluthrin, but not DDT, with resistance associated with elevated levels of esterases [12,32,33]. *An. arabiensis *in Pitoa village near Garoua, northern Cameroon exhibits resistance to permethrin, deltamethrin and DDT associated with elevated levels of esterases and oxidases [13,34-36]. *An. gambiae sensu stricto *in the Kou Valley in northern Bobo-Dioulasso, Burkina Faso exhibit high levels of resistance to pyrethroids associated with high allelic frequencies of the *kdr *mutation among the both the M and S forms [13,37]. *An. gambiae s.s*. in the village of Akron on the periphery of Porto Novo, the administrative capital of Benin [14], are comprised entirely of the M molecular form and are resistant to both pyrethroids and DDT [38]. Resistant *kdr *alleles occur at high frequency in this part of southern Benin and metabolic resistance is also present [38].

In all study sites, unwashed and washed (20 times according to standard protocols [5]) PermaNet^® ^2.0 and 3.0 (Vestergaard Frandsen SA, Switzerland) were compared to an untreated polyester net which served as the negative control for estimating total house entry, survival and feeding rates in the absence of any insecticidal treatment. PermaNet^® ^2.0 is a 100% polyester LLIN [11] coated with a target dose of 1.8 g kg^-1 ^deltamethrin. PermaNet^® ^3.0 is a mosaic-style LLIN designed for higher efficacy against pyrethroid resistant malaria vectors. Its side panels are made of deltamethrin-coated-polyester (with a target dose of 2.8 g kg^-1 ^of deltamethrin), while the top panel is made of monofilament polyethylene fabric into which a higher dose of deltamethrin (4 g kg^-1^) and a synergist, piperonyl butoxide (PBO) (25 g kg^-1^) are incorporated.

### Simulating expected community-level impact based on experimental hut trial results

Mosquito behaviour and survival were modelled as a function of host availability and activity patterns, as well as LLIN properties and coverage exactly as recently described elsewhere [10]. Briefly, this hierarchical approach [6] predicts epidemiologically relevant outcomes such as exposure to transmission (biodemography-epidemiology model) based on the explicit consideration of the activity cycles of wild mosquitoes as they sequentially engage in host-seeking, feeding, resting, oviposition site-seeking, oviposition and back to host-seeking again [39] (behaviour-biodemography model). Detailed consideration of mosquito behaviour and mortality upon encounter with individual hosts allows simulation of the impact of ITNs upon the foraging requirements and risks for mosquito populations at the community level [6]. Impact of LLINs upon malaria transmission intensity is estimated in terms of relative values of the entomologic inoculation rate (EIR) experienced by users and non-users, as well as the community-wide mean, is compared with baseline conditions with no nets [10].

The impact of both LLIN products upon malaria transmission under coverage conditions of 80% use (C_h _= 0.8) was simulated [10] using parameters for overall deterrence (Δ_h, p_), as well as excess mortality occurring before (μ_h, pre_) and after (μ_h, post_) feeding on the human hosts protected by both LLIN products, were derived directly from the raw data. Separate parameter estimates were calculated for both types of net, both before and after washing, consistent with existing WHO guidelines [5] and classification systems that unambiguously assign all mosquitoes caught in an experimental hut trial to one of the following outcomes: deterred, killed before feeding, killed after feeding or fed and survived [10,40]. Correspondingly, and in keeping with standard practice [5], personal protection was calculated as the proportional reduction of blood fed mosquitoes caught.

The availability of individual humans for attack by host seeking mosquitoes, was fixed at 0.0012 attacks per night per person per host-seeking mosquito for the African scenarios in Benin, Burkina Faso and Cameroon [10,41,42]. For the *An. gambiae *populations in Burkina Faso and Benin, the availability of cattle to vectors was set at 0.000025 attacks per head of cattle per night per host-seeking mosquito while for the *An arabiensis *population in Cameroon it was set at 0.0019 attacks per head of cattle per night per host-seeking mosquito as previously described and justified [41,42]. In order to simulate the overwhelmingly zoophagic *An. epiroticus *population in Bac Lieu, Vietnam, the availability of cattle was set at 0.0019, identical to *An. arabiensis*, and the availability of humans calculated by dividing this value by 11.53 based on relative availability measurements [42,43] made in this setting by directly comparing cow and human landing rates [44].

### Statistical analysis

The level of improvement of impact achieved by replacing deltamethrin-only nets with the combination product was assessed treating the proportional reduction of residual transmission as the dependent in a generalized linear model in which washing regime was treated as the only categorical factor, with no washing as the reference group and 20 washes as the test group. The dependent variable was computed as one minus the relative EIR for combination nets compared with deltamethrin-only nets so that the intercept reflects and change relative to the deltamethrin-only nets for fresh, unwashed nets.

## List of abbreviations

IRS: Indoor residual spraying; LLIN: Long-lasting insecticidal net.

## Competing interests

All the phase II experimental hut field trials which were used to parameterize the model, and the modelling study itself, were funded by Vestergaard Frandsen SA. Dr T Knox and Dr H Pates Jamet of Vestergaard Frandsen both provided comments upon the manuscript.

## Authors' contributions

GFK and FOO wrote the mathematical model. GFK extracted the parameter values from the field trial data, implemented the simulations and drafted the manuscript. FOO, RN, MC, JE, RD, AA, SA and VC all contributed to analysis of the experimental hut trials, the interpretation of the model simulation results and to the drafting and finalization of the manuscript. All authors approved the final manuscript.

## Authors' information

All the authors conduct collaborative and sponsored research with a number of pesticide product companies, including Vestergaard Frandsen SA, and explicitly do not recommend any of the particularly products described here.
